# Novel Serotype of Bluetongue Virus, Western North America

**DOI:** 10.3201/eid1904.120347

**Published:** 2013-04

**Authors:** N. James Maclachlan, William C. Wilson, Beate M. Crossley, Christie E. Mayo, Dane C. Jasperson, Richard E. Breitmeyer, Annette M. Whiteford

**Affiliations:** Author affiliations: University of California, Davis, California, USA (N.J. Maclachlan, B.M. Crossley, C.E. Mayo, R.E. Breitmeyer);; US Department of Agriculture–Agricultural Research Service, Manhattan, Kansas, USA (W.C. Wilson, D.C. Jasperson);; California Department of Food and Agriculture, Sacramento, California, USA (A.M. Whiteford)

**Keywords:** Bluetongue virus, viruses, arboviruses, serotype, Ruminants, western North America

**To the Editor:** Bluetongue is an arboviral disease of domestic and wild ruminants characterized by vascular injury that produces widespread edema and tissue necrosis (1). Bluetongue virus (BTV), the causative agent of bluetongue, is the prototype virus of the family *Reoviridae* and the genus *Orbivirus* (2).

BTV occurs throughout temperate and tropical areas of the world coincident with the distribution of vector *Culicoides* spp. midges (3–5). Different midge species transmit different constellations of BTV serotypes in distinct global episystems (3,5). For example, *C. sonorensis* is the principal, if not exclusive, vector of BTV serotypes 10, 11, 13, and 17 in much of North America, whereas *C. insignis* is the major vector of multiple BTV serotypes (including BTV 1–4, 6, 8, 12, 17, 19, 20, and probably others) in the Caribbean basin, Central America, and South America. *C. insignis* is also found in the southeastern United States, and although this species might have recently expanded its range in the region, its distribution in North America remains poorly defined. Serotypes of BTV other than 10, 11, 13, and 17 are found in areas of the United States: BTV-2 was first reported in Florida in 1982. Since 1998, ten additional serotypes (BTV-1, 3, 5, 6, 9, 12, 14, 19, 22, and 24) have been identified in the southeastern United States (6).

Approximately 26 BTV serotypes have been described and the global distribution of BTV has recently been altered (2,4). Coincident with the invasion of novel BTV serotypes into the southeastern United States (6), likely by extension from the adjacent Caribbean basin, multiple BTV serotypes have spread throughout much of continental Europe and parts of the British Isles and Scandinavia, precipitating an economically devastating epidemic (7). Similarly, ongoing surveillance has identified novel BTV serotypes in regions to which it historically has been endemic (e.g., Australia and the Middle East) (2). Climate change may have contributed to this dramatic recent expansion in global distribution of BTV, most notably in Europe (8).

Bluetongue was first described in the late 19th century among sheep brought from Europe to South Africa, and later in North America in ≈1950 (4). Surveillance in western North America since that time has confirmed that only BTV-10, 11, 13 and 17 are present in this region, including our recent intensive surveillance of sentinel cattle on dairy farms throughout California, USA (9,10).

However, during investigation of an outbreak of acute coronitis and ulcerative stomatitis among cattle at a dairy farm in the northern Sacramento Valley in California in August 2010, a blood sample from a heifer was found by using described methods (10) to be positive for BTV by serogroup-specific quantitative reverse transcription PCR (qRT-PCR) but negative by serotype-specific RT-qPCRs for BTV-10, 11, 13, and 17.

Further analysis using additional serotype-specific RT-qPCRs identified virus in the blood sample as BTV-2. BTV was isolated in primary bovine endothelial cells from blood collected from the heifer. Sequence analysis of the serotype-specific L2 gene of the virus isolate confirmed it to be BTV-2 (2), and phylogenetic analyses showed it to be most closely related to a strain of BTV-2 isolated in Florida in 1999 (Figure). However, sequence analysis of the entire genome of the virus from California indicated that it is a reassortant that includes genes from BTV-6 and BTV-2. Specifically, genes encoding the viral protein 1 polymerase and viral protein 3 major core protein segregate with those of the US prototype strain of BTV-6 (isolated in 2006), but other genes are derived from BTV-2. BTV-2 and BTV-6 have been isolated only in the southeastern United States, which indicates translocation within the United States of reassortant BTV-2.

**Figure:**
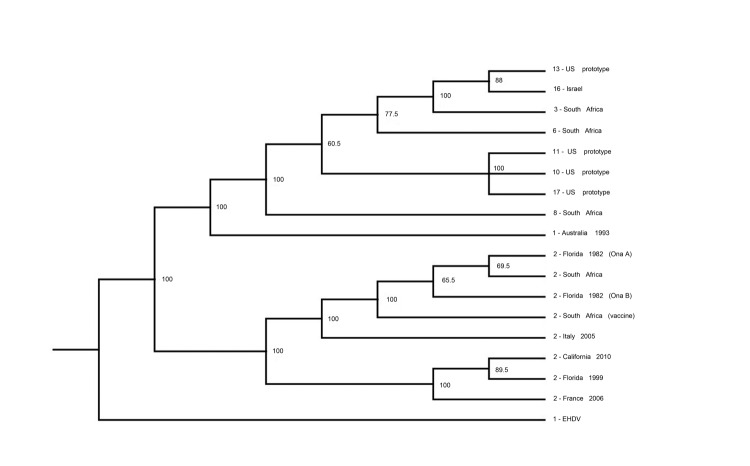
Cladogram comparing the L2 genes of different bluetongue virus (BTV) serotypes and global strains of BTV serotype 2 (BTV-2) with that of a virus isolated in northern California, USA (2-California-2010; GenBank accession nos. JQ822248–JQ822257). Viruses are identified by serotype, country/region of origin, and for isolates of BTV serotype 2, year of identification. Bootstrap percentages are indicated at selected branching points. EHDV1, epizootic hemorrhagic disease of deer virus serotype 1.

How this virus spread to California is not known, and its distribution in the United States is uncertain because there is no comprehensive national BTV surveillance program. However, BTV-2 was not detected previously in California, suggesting that this serotype was recently introduced into the region or that it is uncommon. Identification of this novel BTV serotype in western North America emphasizes the need for ongoing entomologic and livestock surveillance, particularly in light of recent changes in the global distribution and nature of BTV infection (4,6,8).
